# Catastrophic Health Expenditures and Mental Health in Older Chinese People: The Role of Social Health Insurance

**DOI:** 10.1093/geroni/igab046.464

**Published:** 2021-12-17

**Authors:** Wei Yang, Bo Hu

**Affiliations:** 1 King's College London, London, England, United Kingdom; 2 London School of Economics and Political Science, London, England, United Kingdom

## Abstract

Catastrophic health expenditure (CHE) has considerable effects on household living standards, but little is known regarding the impacts of CHE on people’s mental health. Using China as an example, this study examines the association between CHE and mental health and investigates whether and to what extent social health insurance (SHI) can lessen the impacts of CHE on mental health among older people aged over 60 in China. The data come from three waves of the China Health and Retirement Longitudinal Study (CHARLS 2011, 2013, and 2015, N = 13,166). We built fixed-effects quantile regression models to analyse the data. We found that incurring CHE has significantly detrimental effects on older people’s mental health, whereas the SHI demonstrates a protective effect. The observed protective effects of SHI are the strongest among those with relatively mild mental health problems, i.e., people whose CES-D scores are below the 50th percentile. Our findings provide empirical evidence that encourages the integration of psychologically informed approaches in health services. We also urge governments in low- and middle-income countries to consider more generous health financing mechanisms for those with higher healthcare needs.

